# Safflower Alleviates Pulmonary Arterial Hypertension by Inactivating NLRP3: A Combined Approach of Network Pharmacology and Experimental Verification

**DOI:** 10.1111/crj.13826

**Published:** 2024-08-18

**Authors:** Shibiao Ding, Jinyu Cui, Luning Yan, Chuhui Ru, Fei He, Aifeng Chen

**Affiliations:** ^1^ Department of Clinical Laboratory Zhejiang Hospital of Integrated Traditional Chinese and Western Medicine Hangzhou Zhejiang China; ^2^ Department of Respiratory and Critical Care Medicine Zhejiang Hospital of Integrated Traditional Chinese and Western Medicine Hangzhou Zhejiang China

**Keywords:** network pharmacology, NLRP3 inflammation, pulmonary arterial hypertension, quercetin, safflower

## Abstract

**Introduction:**

Traditional Chinese medicinal plant, safflower, shows effective for treating pulmonary arterial hypertension (PAH), yet the underlying mechanisms remain largely unexplored. This study is aimed at exploring the potential molecular mechanisms of safflower in the treatment of PAH.

**Methods:**

Network pharmacology approach and molecular docking were applied to identify the core active compounds, therapeutic targets, and potential signaling pathways of safflower against PAH. Meanwhile, high‐performance liquid chromatography (HPLC) assay was performed to determine the core compounds from safflower. Further, the mechanism of action of safflower on PAH was verified by in vivo and in vitro experiments.

**Results:**

A total of 15 active compounds and 177 targets were screened from safflower against PAH. Enrichment analysis indicated that these therapeutic targets were mainly involved in multiple key pathways, such as TNF signaling pathway and Th17 cell differentiation. Notably, molecular docking revealed that quercetin (core compound in safflower) displayed highest binding capacity with NLRP3. In vivo, safflower exerted therapeutic effects on PAH by inhibiting right ventricular hypertrophy, inflammatory factor release, and pulmonary vascular remodeling. Mechanistically, it significantly reduced the expression of proangiogenesis‐related factors (MMP‐2, MMP‐9, Collagen 1, and Collagen 3) and NLRP3 inflammasome components (NLRP3, ASC, and Caspase‐1) in PAH model. Similarly, these results were observed in vitro. Besides, we further confirmed that NLRP3 inhibitor had the same therapeutic effect as safflower in vitro.

**Conclusion:**

Our findings suggest that safflower mitigates PAH primarily by inhibiting NLRP3 inflammasome activation. This provides novel insights into the potential use of safflower as an alternative therapeutic approach for PAH.

## Graphical abstract

Integrating network pharmacology, molecular docking, and experimental validation to identify the effects of safflower against pulmonary arterial hypertension.

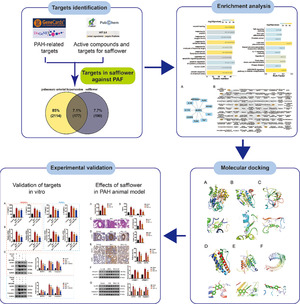

## 1 Introduction

Pulmonary arterial hypertension (PAH) is a serious life‐threatening cardiovascular disorder characterized by the progressive remodeling of the pulmonary arteries, leading to increased pulmonary vascular resistance, right heart failure, and ultimately death [1]. The global prevalence of PAH is estimated to be 0.4–1.4 cases per 100 000 persons, with a higher incidence among females [2]. The pathogenesis of PAH is believed to be influenced by a combination of external and internal environmental factors, including infections, diet, internal imbalances, and genetic predispositions [3]. Despite considerable advancements in therapeutic approaches that have significantly improved clinical symptoms of PAH, the progression of the disease remains inevitable for most patients [4]. This observation is particularly pronounced in developing countries, where a majority of PAH patients still succumb to the disease even after receiving current pharmacological treatments, with a mere 5‐year survival rate of 59% [5]. Therefore, there is an urgent need for novel therapies that can ameliorate the progression of PAH and improve patient survival.

Renowned for its holistic and multitargeted approach, traditional Chinese medicine (TCM) offers a promising alternative for addressing complex diseases like [6]. Among various TCM, safflower ( *Carthamus tinctorius* L.), a medicinal plant of the Asteraceae family, is recognized for its various pharmacological properties, such as antioxidant, lipid‐lowering, and anti‐inflammatory activities [7]. In clinical practice, safflower is primarily employed in the prevention and treatment of vascular‐related diseases, including cardiovascular diseases and occlusive cerebrovascular diseases [8, 9]. Hydroxysafflor yellow A, a compound extracted from safflower, has been demonstrated to alleviate hypoxia‐induced pulmonary artery remodeling and reverse right ventricular hypertrophy in rats [10]. Previous study has also confirmed that safflower injection (SI) significantly inhibits pulmonary artery reconstruction in rats with PAH induced by *Veratrum nigrum* alkaloids [11]. Nevertheless, the targets, active compounds, and action mechanisms of safflower in the treatment of PAH warrant further investigation.

Motivated by these knowledge gaps, our research is aimed at dissecting the intricate mechanisms of safflower in PAH treatment through a network pharmacology approach. Furthermore, we performed molecular docking to investigate the binding efficiency of the active compounds with their potential targets and then supplemented by in vivo and in vitro experiments for verification. This comprehensive exploration will help in understanding the role of safflower in PAH, providing new avenues for the development of targeted therapies for PAH.

## 2 Materials and Methods

### 2.1 Retrieval of PAH‐Related Targets From Databases

The GeneCards (http://www.genecards.org) [12] and DisGeNET (http://www.disgenet.org) [13] databases were queried using “pulmonary arterial hypertension” as a keyword to identify PAH‐related targets. Following the consolidation of retrieved data, duplicate entries were eliminated.

### 2.2 Identification of Active Compounds and Targets of Safflower

Active compounds of safflower were sourced from the Traditional Chinese Medicine Systems Pharmacology (TCMSP) database (https://old.tcmsp‐e.com/tcmsp.php) [14]. In addition, compound names were validated with the PubChem database (https://pubchem.ncbi.nlm.nih.gov/). To evaluate drug‐likeness (DL), the physicochemical properties of these compounds were compared with marketed drugs, adopting the criterion oral bioavailability (OB) ≥ 20% and DL ≥ 0.1 as defined by Xu et al. and Tao et al. [15, 16]. Subsequent to this, the targets of these compounds were determined via the HIT database (Herb Ingredients' Targets, http://www.badd‐cao.net:2345/) [17]. Active compounds devoid of targets within the database were removed, and the remaining targets were consolidated.

### 2.3 Identification of Safflower's Targets Against PAH

To identify the potential targets of safflower against PAH, we conducted an intersection of the PAH‐related targets and active component‐related targets using VennDiagram (Version 1.7.3) [18].

### 2.4 Construction of the Protein–Protein Interaction (PPI) network

Intersected genes underwent PPI network analysis via the STRING database [19]. The selection criteria were score_cutoff > 0.7 and size_cutoff < 10. PPI network was visualized using Cytoscape software.

### 2.5 Function Enrichment Analysis

To explore the biological functions of potential targets in PPI network, Gene Ontology (GO) and KEGG enrichment analyses were performed using the clusterProfiler tool (Version 4.4.2) [20].

### 2.6 High‐Performance Liquid Chromatography (HPLC) Analysis

The SI (Chinese medicine No. Z14020783) was purchased from Yabao Pharmaceutical Group Co. Ltd. (Shanxi, China). HPLC analysis was undertaken to quantify the active compounds in SI. The control solution was meticulously prepared by dissolving precisely weighed standards of hydroxysafflor yellow A (Catalog No. B20968; Shanghai Yuanye Bio‐Technology Co., Ltd., Shanghai, China) and quercetin (Catalog No. B20527; Shanghai Yuanye Bio‐Technology Co., Ltd.) in methanol to attain concentrations of 101.2 and 108.4 μg/mL, respectively. The prepared solution was subsequently filtered using a 0.45 μm pore size membrane filter.

For the sample preparation, an exact 1 mL aliquot of SI was drawn and diluted to a total volume of 10 mL using 25% methanol. The sample was then subjected to filtration through a 0.45 μm pore size membrane filter.

Chromatographic separation was accomplished utilizing an Agilent 1260 HPLC system, outfitted with a NanoChrom ChromCore C18‐AC column (5 μm, 4.6 × 250 mm). The operational flow rate was set at 1 mL/min, and the injection volume was maintained at 5 μL. The mobile phase consisted of methanol (Component A) and a 0.1% aqueous solution of phosphoric acid (Component B). The column temperature was stabilized at 30°C, and ultraviolet detection was carried out at a wavelength of 280 nm. The gradient elution program commenced with a mobile phase ratio of 90%–60% B from 0 to 10 min, transitioning to 60%–56% B from 10 to 25 min, followed by a shift to 56%–40% B from 25 to 60 min, and concluding with a 40%–15% B gradient from 60 to 70 min.

### 2.7 Selection of Candidate Genes for Molecular Docking

We performed an intersection analysis for each active compound's targets and the intersected genes. The active compound with the most shared targets was identified as the core compound of safflower. The NLRP3 gene was recognized as associated with PAH from the literature [21]. Nine genes that interact with NLRP3 were discerned from the PPI network. These 10 key targets, including NLRP3 and the nine interacting genes, along with the core compound of safflower, were integrated into subsequent molecular docking analysis.

### 2.8 Molecular Docking Analysis

The molecular docking procedure was utilized to assess the binding affinity between the identified key targets and safflower's core compounds. The structural data for target proteins and core components (in mol2 format) were sourced from the RCSB protein database (http://www.pdb.org/) and ZINC database (https://zinc.docking.org/), respectively. Using Open Babel GUI software, the downloaded protein and core compound files were converted into PDBQT format. Protein preparation steps, such as removal of original ligands, water, and addition of hydrogen, as well as amino acid optimization and charge calculations, were conducted using PyMOL and AutoDock 1.5.6 software [22]. Finally, molecular docking was performed using AutoDock 1.5.6, and the results were visualized with PyMOL.

### 2.9 Animal Modeling

Eighteen male Sprague Dawley rats, aged 8 weeks and weighing 230–250 g, were acquired from Xiamen University's Experimental Animal Center. These animals were housed in a controlled environment, with a 20°C–26°C temperature and 50% ± 5% relative humidity. The rats experienced a 12‐h light/dark cycle and had unrestricted access to food and water.

After 1 week of acclimatization, the rats were randomly divided into three groups (*n* = 6 per group): control, monocrotaline (MCT, PAH model), and MCT + SI. To induce a model of PAH, the MCT and MCT + SI groups received a single dose of subcutaneous injections of MCT (60 mg/kg) followed the method described by Chang et al. [23]. The control group received subcutaneous injections of an equivalent volume of saline on the first day. Following the initial treatment, the MCT + SI group received intraperitoneal injections of a SI (2 mL/kg/day) for 20 consecutive days [11]. Equivalent volumes of saline were injected in the control and PAH groups. At the end of the treatment period, the rats were anesthetized with an intraperitoneal injection of sodium pentobarbital (40 mg/kg), and then, blood samples were collected from the heart, as well as tissue samples from the lungs and heart were harvested. The animals were subsequently euthanized.

### 2.10 Assessment of Right Ventricular Function

Following euthanasia, the hearts were carefully removed from the rats. The left and right atria, along with the roots of the major blood vessels, were excised. The right ventricle was carefully separated, and then, the remaining left ventricle, septum, and the isolated right ventricle were blotted dry with filter paper and weighed. The right ventricular hypertrophy index (RVHI) was then calculated using the following formula: RVHI = (right ventricle) RV/((left ventricle) LV + (interventricular septum) S).

### 2.11 Hematoxylin and Eosin (H&E) Staining

The evaluation of pulmonary vascular remodeling in lung tissue samples was carried out using H&E staining. Lung tissues were first fixed in 10% formaldehyde for 48 h before being thoroughly rinsed and dehydrated through immersion in a series of ethanol solutions. Tissue clearing was performed by immersing the samples in a 50% ethanol‐50% xylene mixture and subsequently in 100% xylene. The tissues were then infiltrated with paraffin wax, embedded into blocks, and chilled at −20°C before sectioning into 4 μm slices. Prepared slides were baked, dewaxed in xylene, and rehydrated before being stained with hematoxylin. The tissues were then stained with eosin, dehydrated, and cleared with xylene. Slides were mounted using neutral resin and heated. Finally, microscopic images (Olympus, Japan) were captured for tissue observation.

### 2.12 Immunohistochemistry

In the immunohistochemical detection of matrix metalloproteinase‐2 (MMP2) and MMP9 in lung tissue, paraffin‐embedded tissue sections were prepared and deparaffinized. Following this, antigen retrieval was performed using a high‐pressure cooking method with citric acid repair solution. To block endogenous peroxidase activity, a 3% hydrogen peroxide solution was used. The slices were then incubated with primary antibodies against α‐smooth muscle actin (α‐SMA; Affinity; AF1032; 1:100), MMP2 (Abcam, UK; ab86607; 1:250), and MMP9 (Abcam; ab76003; 1:100), followed by overnight incubation at 4°C. A second incubation with secondary antibodies (Abcam; ab205718; 1:2000) was conducted the following day. The chromogenic reaction was carried out with DAB solution, controlled to optimal results, and stopped by rinsing with distilled water. Finally, differentiation was achieved with 1% hydrochloric acid alcohol and monitored under a microscope (Olympus).

### 2.13 Primary Cell Isolation and Treatment

The primary pulmonary artery smooth muscle cells (PASMCs) and pulmonary artery endothelial cells (PAECs) were isolated from pulmonary arteries of 8‐week‐old male Sprague Dawley rats and cultured as previously reported [24, 25]. The identification of PASMCs and PAECs was determined by immunostaining for α‐SMA (Affinity, OH, United States; AF1032; 1:200) and von Willebrand factor (vWF; Affinity; AF3000; 1:300), respectively. Immunofluorescence staining confirmed that the cultured cells contained over 90% PASMCs and 95% PAECs (Figure S1).

PASMCs and PAECs were pretreated with 10 μmol/L NLRP3 inhibitor MCC950 for 30 min, respectively [26]. Then, cells were treated with MCT (1 μmol/mL) for 24 h. MCT‐treated cells were maintained with SI (200 μg/mL) for 48 h. The dose of reagent was determined according to our previous study [27].

### 2.14 ELISA

The concentrations of proinflammatory cytokines IL‐1β (ml037361) and IL‐18 (ml002816) in the serum or cells were measured using corresponding ELISA kits (Mlbio, Shanghai, China) according to the manufacturer's instructions.

### 2.15 Quantitative Real‐Time PCR (qRT‐PCR)

The mRNA expression of MMP2 and MMP9 in cells was measured as a reported method [28]. In brief, total RNA was extracted from the cells using TRIzol reagent (Invitrogen, #15596018, CA, United States), followed by reverse transcription to obtain cDNA using FastKing‐RT SuperMix kit (TIANGEN, #KR118‐02, Beijing, China). Finally, qRT‐PCR analysis was performed with the help of SYBR Green PCR Master Mix kit (Lifeint, #A4004M, Xiamen, China) and real‐time PCR system (Applied Biosystems, California, United States). The relative expression of target genes was calculated using the 2^−ΔΔCt^ method with GAPDH as the internal reference. The gene sequences used for qRT‐PCR were as follows: MMP2 forward 5′‐GGTGGCAATGGAGATGGACA‐3′ and reverse 5′‐CCCGGTCATAATCCTCGGTG‐3′, MMP9 forward 5′‐GATCCCCAGAGCGTTACTCG‐3′ and reverse 5′‐GTTGTGGAAACTCACACGCC‐3′, and GAPDH forward 5′‐GCGAGATCC CGCTAA CATCA‐3′ and reverse 5′‐CTCGTGGTTCACACCCATCA‐3′.

### 2.16 Western Blotting

Total proteins of lung tissues or cells were extracted using RIPA lysis buffer (Beyotime, Shanghai, China), and protein concentration was confirmed using a BCA protein assay kit (Beyotime). An equivalent of 25 μg of protein was prepared for SDS‐PAGE (Bio‐Rad, CA, United States) and then transferred onto a PVDF membrane. Nonspecific binding was prevented by blocking the membrane with 5% nonfat milk. The blocked membranes were then subjected to incubation with primary antibodies against Collagen 1 (Affinity; AF7001; 1:500), Collagen 3 (Affinity; AF5457; 1:1000), NLRP3 (Affinity; DF15549; 1:2000), Caspase‐1 (Affinity; AF5418; 1:2000), ASC (Affinity; DF6304; 1:2000), and GAPDH (Cell Signaling Technology, MA, United States; 2118; 1:1000). Following this, a secondary antibody was applied at a 1:2000 dilution. Chemiluminescent detection was carried out using an ECL reagent (Applygen, Beijing, China).

### 2.17 Data Analysis

All data are presented as mean ± standard deviation. Differences between groups were evaluated using one‐way analysis of variance. All statistical analyses were performed using GraphPad Prism 7.0 software. *p* < 0.05 was considered statistically significant.

## 3 Results

### 3.1 Identification of Safflower's Targets for PAH

**FIGURE 1:**
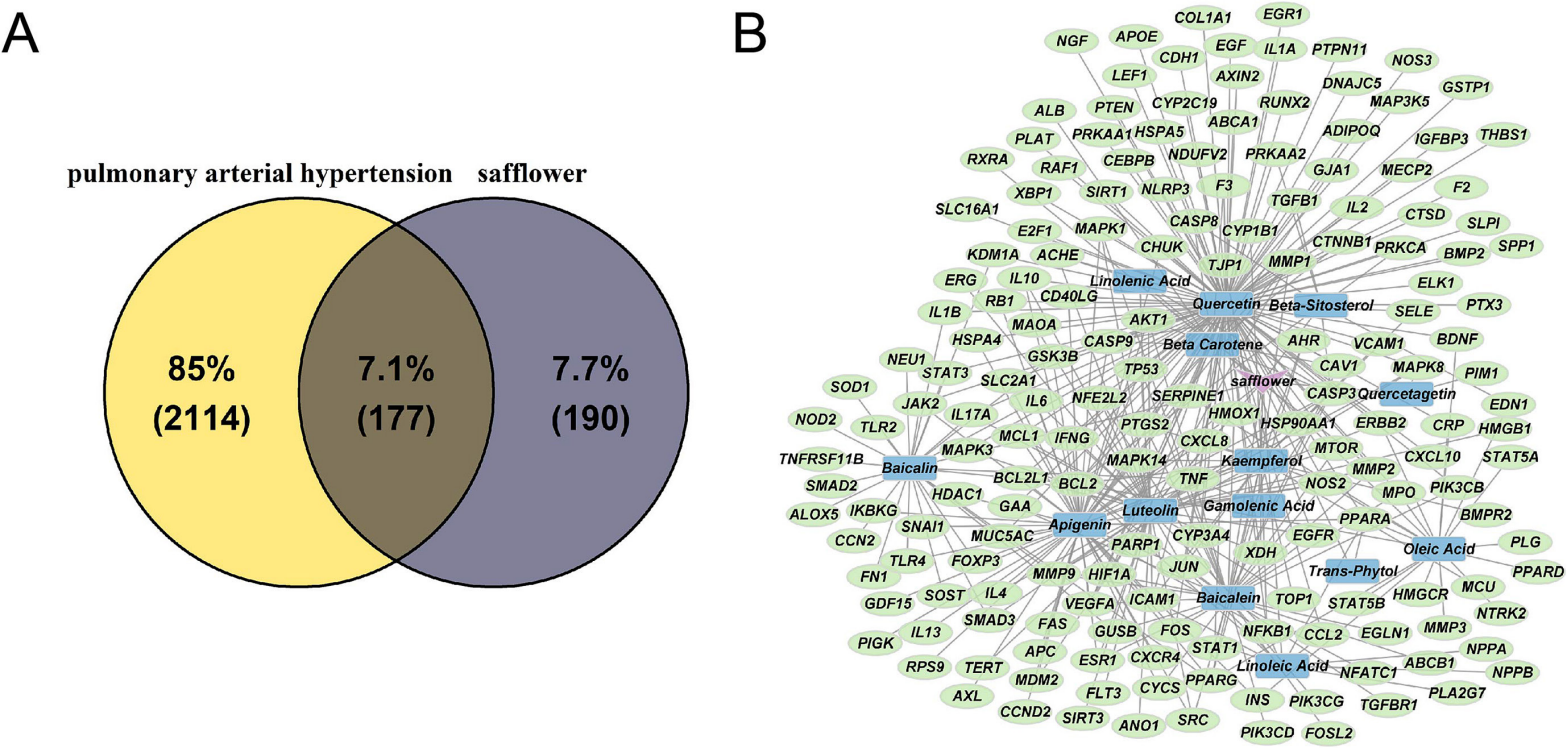
Identification of active compounds and potential targets of safflower for pulmonary arterial hypertension (PAH) management. (A) A Venn diagram showcasing the intersection of 177 shared targets between PAH and safflower‐related targets. (B) The drug‐compound‐common target network. Active compounds are denoted in blue, drugs in purple, and common targets in green.

We identified 2291 PAH‐related targets using GeneCards and DisGeNET databases. Moreover, 15 active compounds with 367 target genes were identified from safflower. A Venn diagram indicated an intersection of 177 common targets between PAH‐ and safflower‐related targets (Figure 1A). We then created a network of drug‐compound‐common targets using Cytoscape software, where each edge represents an interaction between compounds or targets (Figure 1B).

### 3.2 PPI Network

**FIGURE 2:**
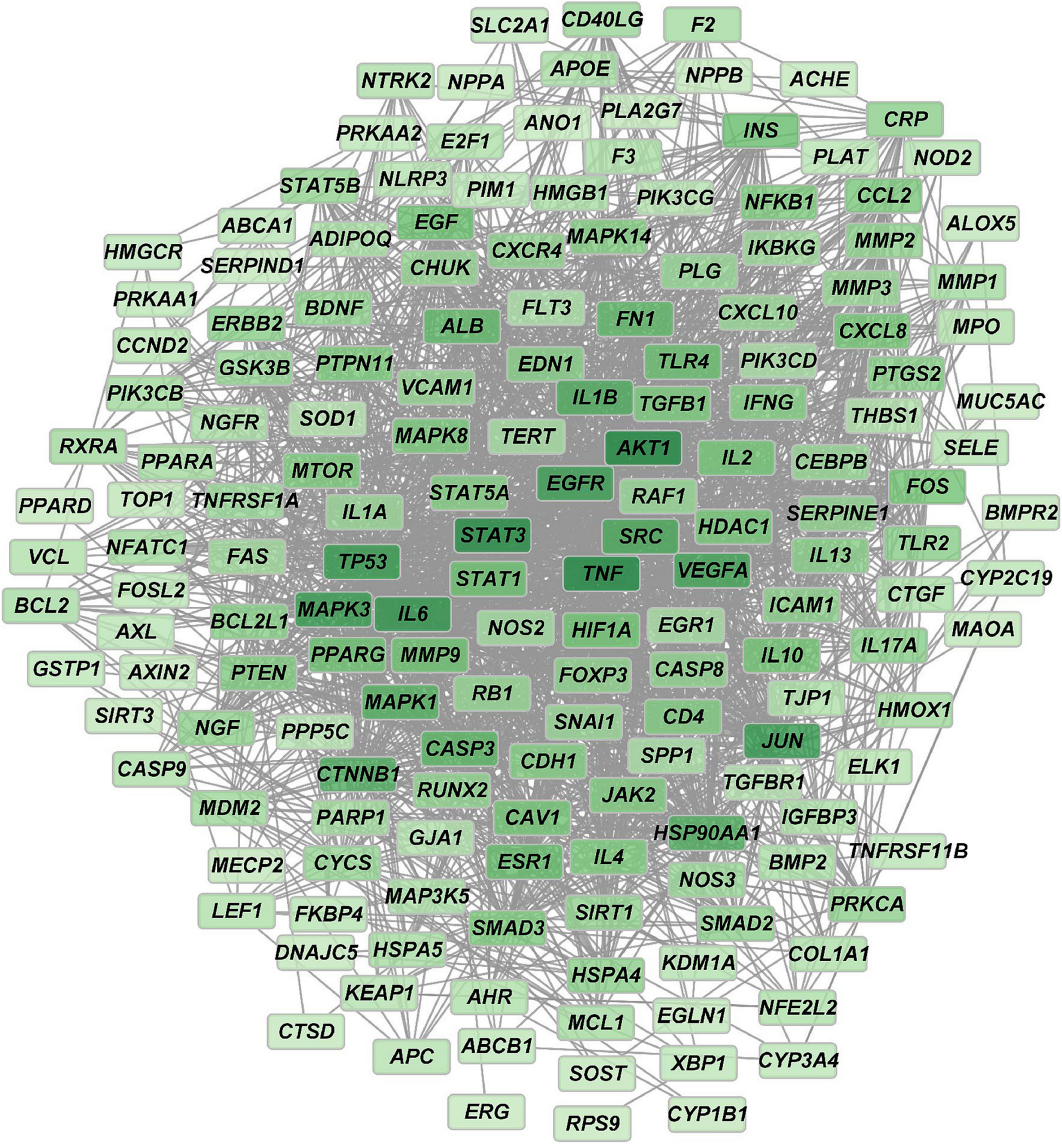
Protein–protein interaction (PPI) network. The network visualizes interactions among the 177 shared targets.

A PPI network was developed using the STRING database, illustrating the interactions among the 177 common targets. Based on a score cutoff > 0.7 and size cutoff < 10, we found 1977 interaction relationships among these common targets (Figure 2).

### 3.3 Biological Function Enrichment Analysis

**FIGURE 3:**
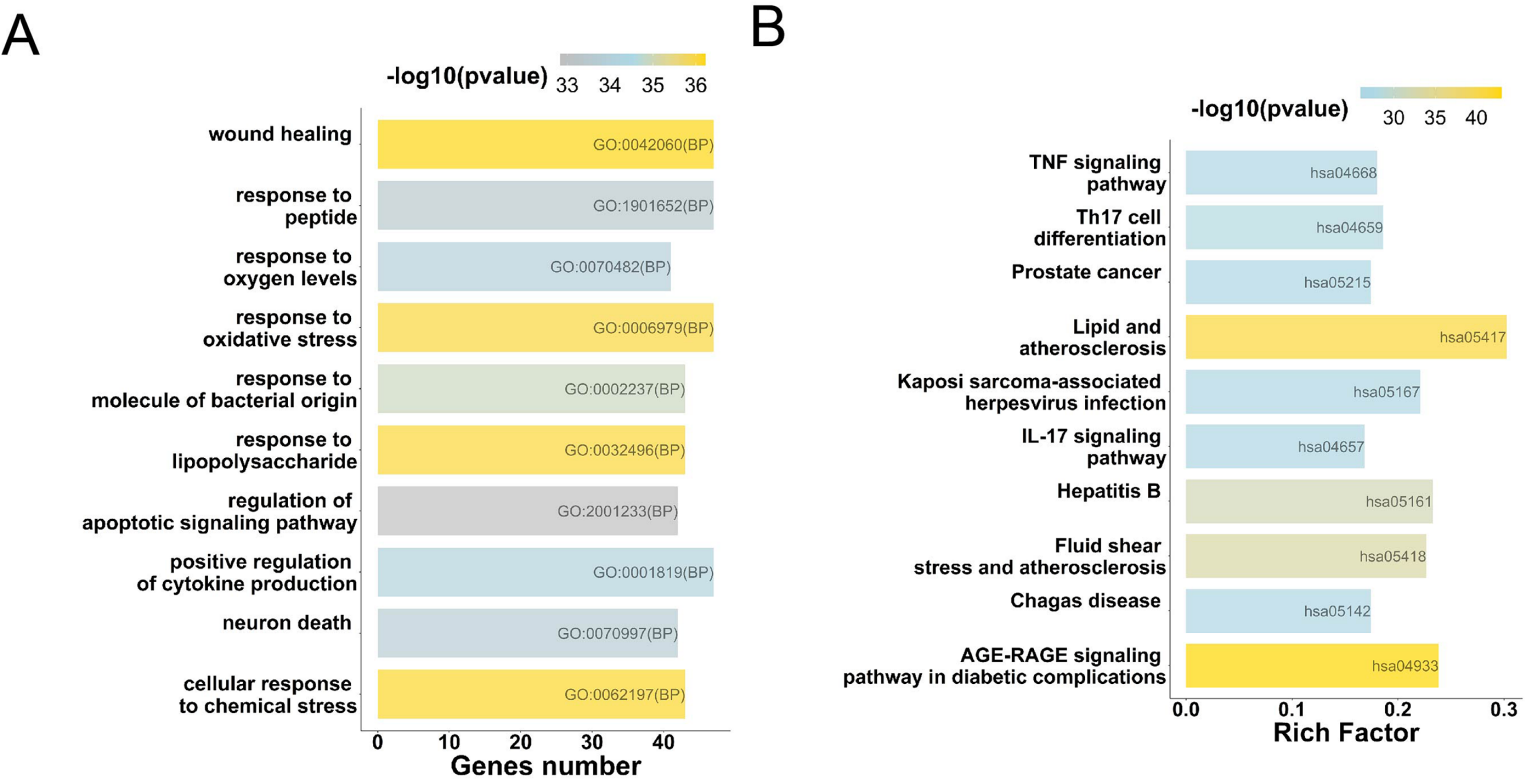
Biological function enrichment analysis. (A) The Top 10 Gene Ontology (GO) biological processes highlight the primary biological activities influenced by safflower in PAH management. (B) The Top 10 KEGG pathways underscore the essential pathways that safflower engages to combat PAH.

Function enrichment analysis revealed that 177 common targets were significantly enriched in 2376 GO terms and 166 KEGG pathways. The Top 10 GO biological processes showed that wound healing, response to peptide and oxidative stress, and positive regulation of cytokine production are primarily involved in safflower's action against PAH (Figure 3A). The Top 10 KEGG pathways indicated that safflower mainly acts against PAH via the TNF signaling pathway, Th17 cell differentiation, and prostate cancer, as well as lipid and atherosclerosis pathways (Figure 3B).

### 3.4 Molecular Docking Analysis

**FIGURE 4:**
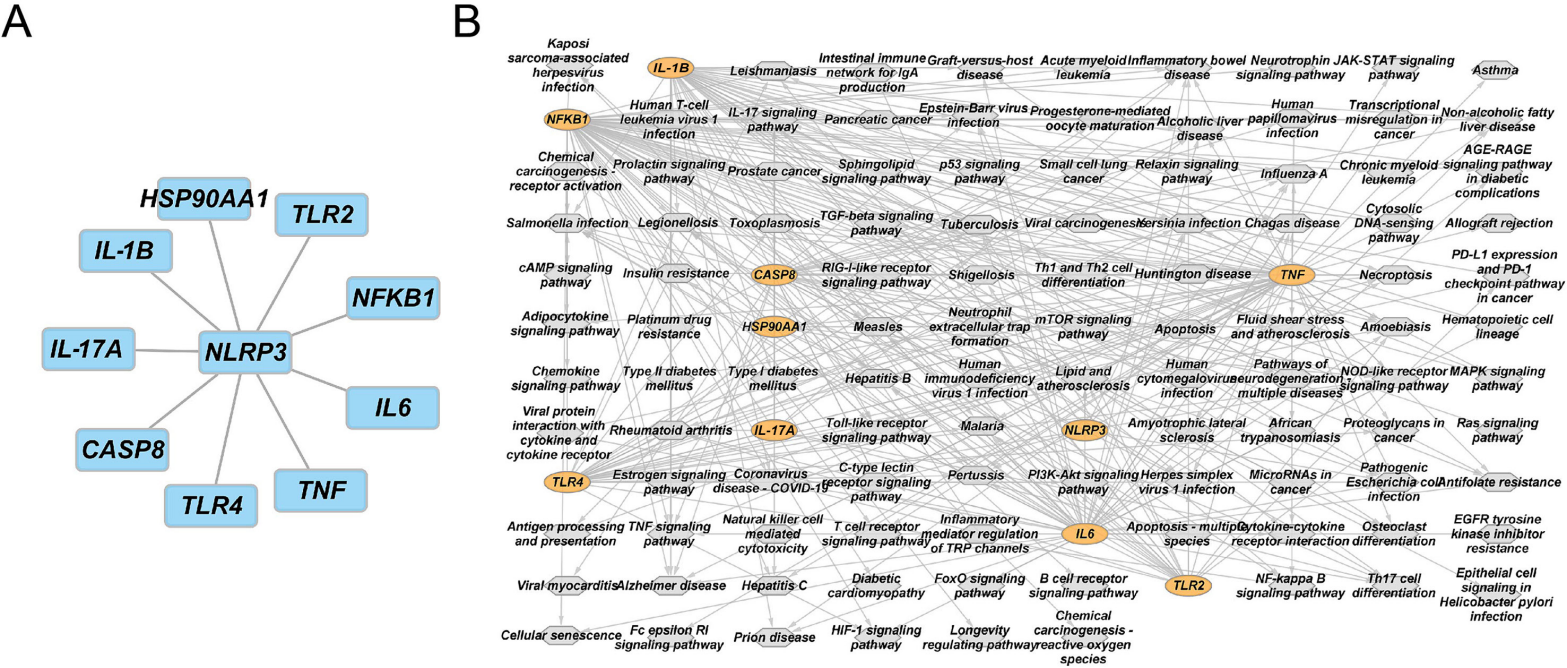
Core compounds and target identification and network construction. (A) PPI network depicting NLRP3 interactions. (B) The target‐pathway network, highlighting the primary pathways involving the core targets.

HPLC analysis demonstrated that hydroxysafflor yellow A and quercetin constitute the principal active constituents in the SI. Hydroxysafflor yellow A displayed a peak elution time of 11.141 min and a concentration of 289.93281 μg/mL, while quercetin manifested a peak elution time of 27.099 min and a concentration of 8.41601 μg/mL (Figure S2). Quercetin emerged as a key safflower compound for PAH, given it shared the most targets (144 out of 177). Literature review revealed NLRP3's critical role in PAH, along with TLR2, NFKB1, IL‐1B, HSP90AA1, TNF, TLR4, IL6, IL‐17A, and CASP8, which closely interacted with NLRP3 (Figure 4A). These genes, including NLRP3, could be core safflower targets for PAH. The target‐pathway network further indicated that these core targets mainly involve the PI3K‐Akt signaling pathway, apoptosis, and MAPK signaling pathway (Figure 4B).

**FIGURE 5:**
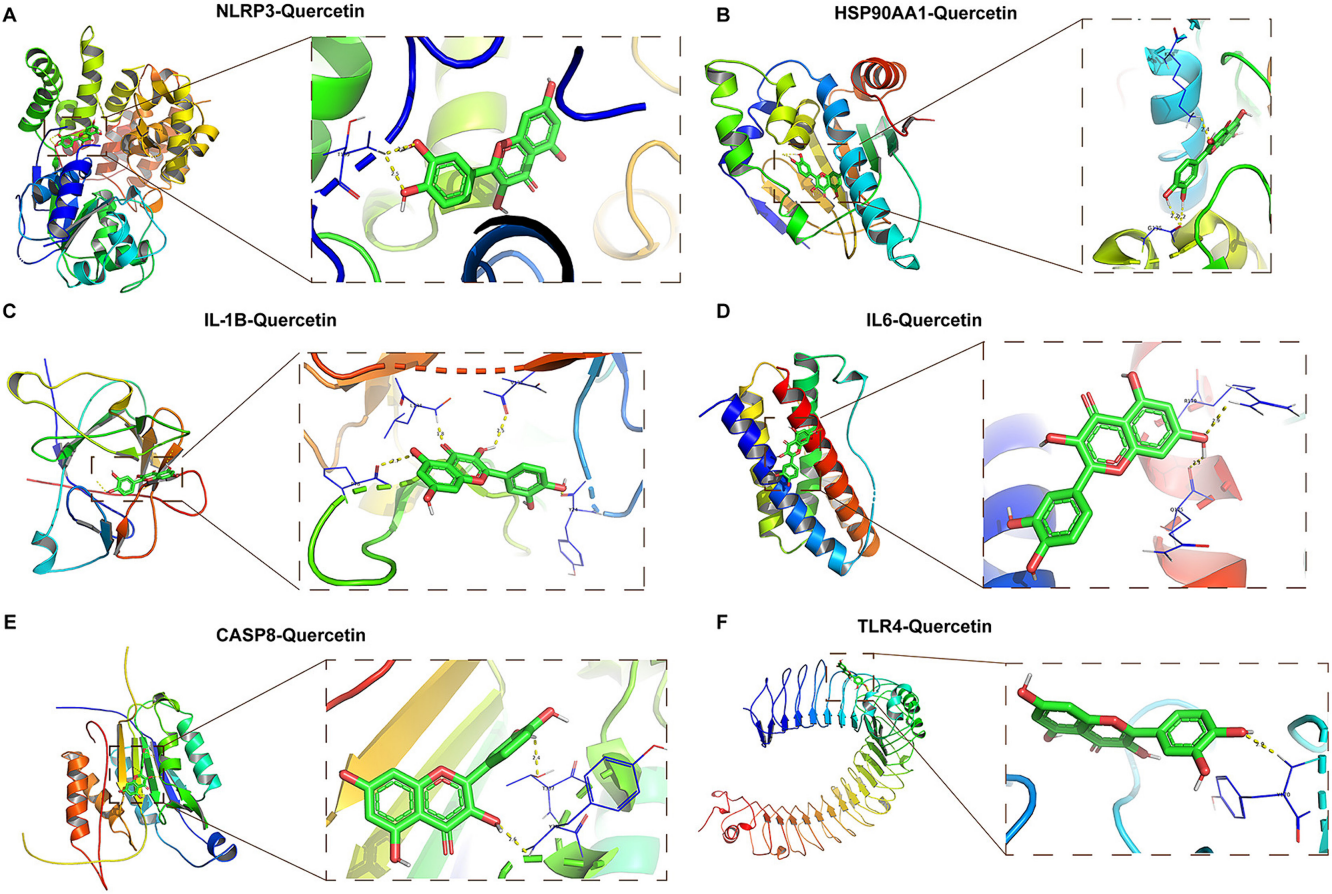
Molecular docking analysis illustrates potential binding modes and the reliability of interactions between core compounds and targets of safflower for PAH treatment.

We investigated potential binding modes and the reliability of interactions between core compounds and core targets using molecular docking. Compounds with affinity less than −5.0 kcal/mol were considered as having strong binding [29]. Accordingly, quercetin showed strong binding with NLRP3 (−8.9 kcal/mol), HSP90AA1 (−7.6 kcal/mol), IL‐1B (−7.1 kcal/mol), IL6 (−7.1 kcal/mol), CASP8 (−7 kcal/mol), and TLR4 (−6.8 kcal/mol) (Figure 5A–F).

### 3.5 Experimental Verification

**FIGURE 6:**
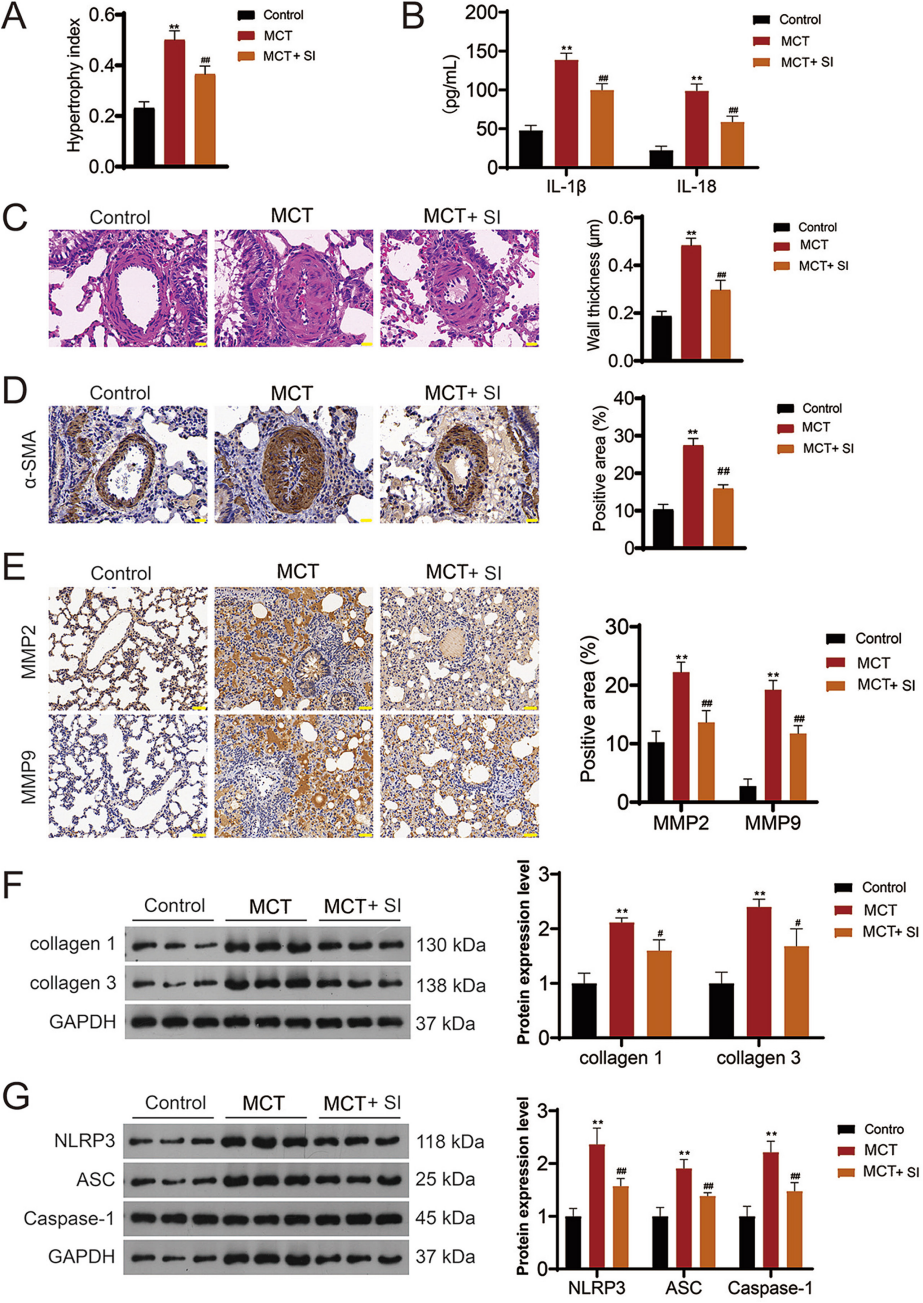
Experimental validation using a monocrotaline (MCT)‐induced PAH rat model. (A) The right ventricular hypertrophy index in the PAH rat model following safflower injection (SI) treatment. (B) Expression levels of inflammatory markers (IL‐1β and IL‐18) in lung tissues post‐SI treatment. (C) Hematoxylin and eosin staining of lung tissue across different experimental groups; scale bar: 50 μm. (D, E) Immunohistochemical analysis of α‐SMA and matrix metalloproteinase‐2 and matrix metalloproteinase‐9 (MMP2 and MMP9) post‐SI treatment; scale bar: 50 μm. (F) Expression levels of Collagen 1 and Collagen 3 post‐SI treatment. (G) Western blot analysis of NLRP3 inflammasome components (NLRP3, ASC, and Caspase‐1) post‐SI treatment. **p < 0.01 versus the control group; ^#^ *p* < 0.05 and ^##^ *p* < 0.01 versus the PAH group.

To validate the findings from our network pharmacology analysis, we developed a MCT‐induced PAH rat model. As demonstrated in Figure 6A, the MCT group exhibited a significant rise in the RVHI compared to the control group. Remarkably, treatment with SI substantially mitigated this increase in the MCT rats (*p* < 0.01). The inclusion of SI significantly downregulated the expression levels of inflammatory markers (IL‐1β and IL‐18) in the lung tissues of MCT rats (*p* < 0.01; Figure 6B). H&E staining further confirmed intact lung tissue morphology, continuous endothelial cells, and smooth pulmonary vascular walls with no unusual thickening in the control group. In contrast, the MCT group showed evident pulmonary vascular wall thickening and lumen narrowing. However, SI treatment notably ameliorated pulmonary vascular remodeling in the MCT rats (Figure 6C). α‐SMA, a marker commonly used to identify smooth muscle cells, was increased in the lung tissues of MCT rats compared to control rats (*p* < 0.01), but SI reduced its level (*p* < 0.01; Figure 6D). Additionally, SI addition significantly downregulated the expression level proangiogenic‐related factors (Collagen 1, Collagen 3, MMP2, and MMP9) in the lung tissues of MCT rats (Figure 6E,F). Lastly, the western blot results demonstrated that SI effectively attenuated the MCT‐induced upregulation of NLRP3 inflammasome components (NLRP3, ASC, and Caspase‐1) (*p* < 0.01; Figure 6G).

**FIGURE 7:**
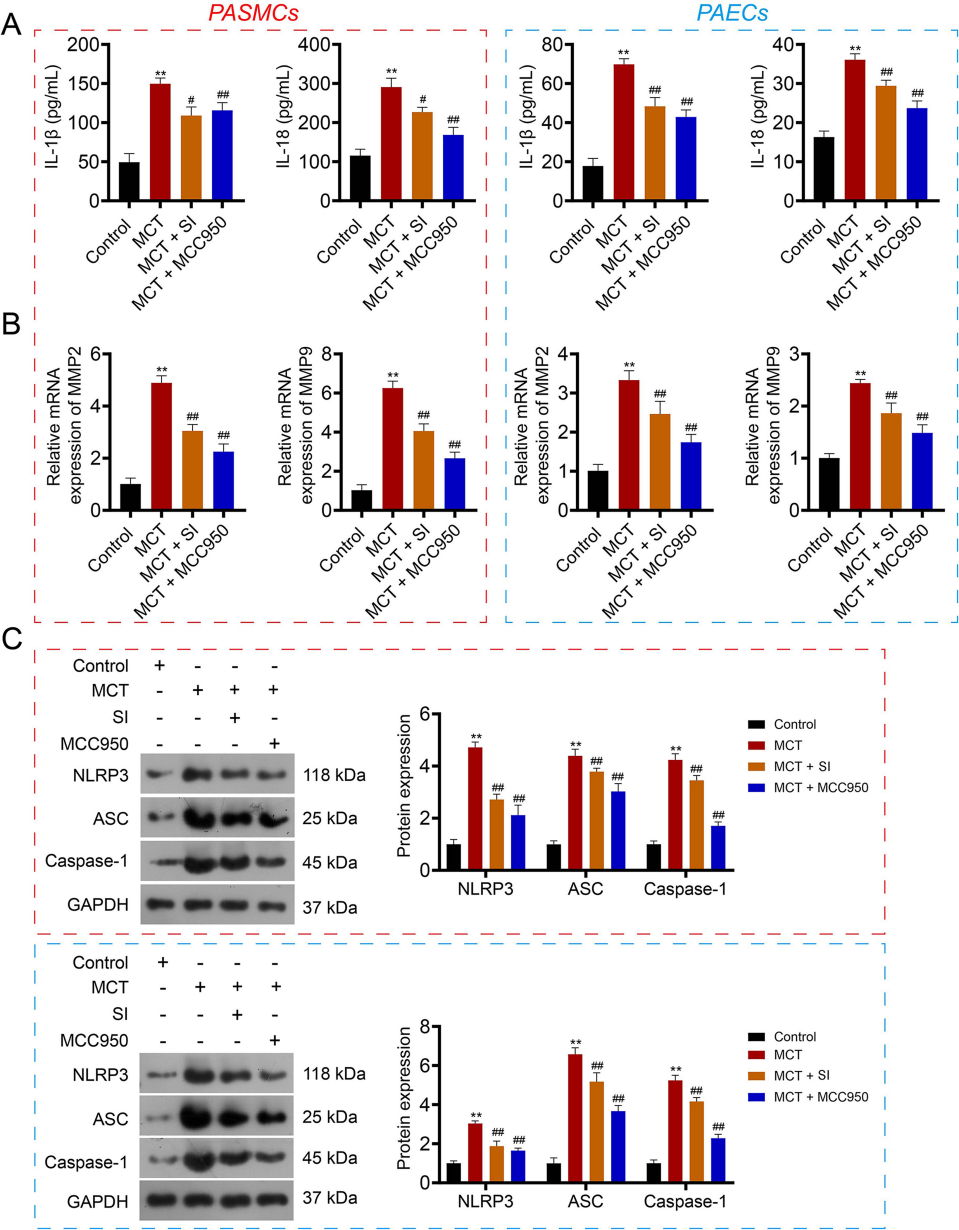
Validation of the inhibitory effects of safflower on NLRP3 inflammasome activation in primary pulmonary artery smooth muscle cells (PASMCs) and pulmonary artery endothelial cells (PAECs). (A) ELISA results indicating the expression levels of IL‐1β and IL‐18 posttreatment with SI or the NLRP3 inhibitor MCC950. (B) Quantitative real‐time PCR (qRT‐PCR) results showcasing expression levels of MMP2 and MMP9 posttreatment with SI or MCC950. (C) Western blot analysis demonstrating expression levels of NLRP3 inflammasome components (NLRP3, ASC, and Caspase‐1) posttreatment with SI or MCC950. **p < 0.01 versus the control group; ^#^ *p* < 0.05 and ^##^ *p* < 0.01 versus the MCT group.

To substantiate that safflower could inhibit NLRP3 inflammasome activation in lung tissues, primary PASMCs and PAECs were treated with MCT alongside SI or the NLRP3 inhibitor MCC950. Both ELISA and qRT‐PCR results indicated that SI and MCC950 treatment significantly reversed MCT‐induced upregulation of IL‐1β, IL‐18, MMP2, and MMP9 (*p* < 0.05; Figure 7A,B). Furthermore, SI and MCC950 treatment effectively curbed the MCT‐induced rise in the expression of NLRP3, ASC, and Caspase‐1 (*p* < 0.01; Figure 7C).

## 4 Discussion

PAH remains a pressing health issue worldwide due to its high morbidity/mortality and the absence of definitive treatments [30]. Previous study has found that SI can alleviate PAH via improving pulmonary artery remodeling [11], but the underlying mechanisms are largely unknown. Hence, we leveraged a network pharmacology approach, molecular docking, and experimental validation to unveil the underlying mechanisms of action of safflower against PAH.

Through network pharmacology screening, quercetin was found to be the core compound in safflower, which was confirmed by HPLC analysis and previous study [31]. Quercetin is a natural dietary flavonoid with a wide range of pharmacological effects, such as anti‐inflammatory, antioxidant, and anticancer activities [32]. The researchers have found that quercetin slows the progression of MCT‐induced PAH in rats by multiple mechanisms that may involve the TrkA/AKT and Erk1/2 signaling pathways [33, 34]. In addition, we predicted that 177 proteins may be key targets of safflower against PAH, and their enrichment pathways were mainly involved in the TNF signaling pathway and Th17 cell differentiation. It has been shown that TNF‐α can further promote the development of PAH by reducing the BMPR2 expression (a key factor in lung remodeling) in PASMCs, and baicalin can regulate the TNF‐α signaling pathway to prevent experimental PAH [35]. Th17 cells are highly proinflammatory and are a subpopulation of effector T cells that produce IL‐17 [36]. Notably, IL‐17 plays an important role in PAH and is correlated with the severity of the disease [37]. Together, we speculate that safflower may exert therapeutic effects by affecting TNF signaling and Th17 cell differentiation pathways.

Further molecular docking revealed that the core compound quercetin had a good binding affinity with several key targets, including NLRP3, HSP90AA1, IL‐1B, IL6, CASP8, and TLR4. Activation of NLRP3 in PAH contributes to the inflammatory response and vascular proliferation [38]. HSP90AA1 is abnormal expressed in PAH patients and involved in airway inflammatory response, which may be a potential target for PAH treatment [39]. Inflammatory cytokines such as IL‐1B and IL6 are overexpressed in PAH, and tanshinone IIA protects broilers from PAH by regulating these factors [40]. TLR4 is expressed on platelets and mediates immune response. Previous study has indicated that TLR4‐deficient mice may reduce PAH susceptibility by attenuating the inflammatory response of the pulmonary vasculature to chronic hypoxia [41]. However, the link between CASP8 and PAH has not been reported. These evidences indicate that these key targets are involved in PAH pathogenesis, further confirming that safflower may reverse PAH through modulating the expression of these targets.

We also utilized in vivo and in vitro experiments to validate the network pharmacology results. In vivo research confirmed that safflower ameliorates PAH symptoms primarily by regulating inflammation and pulmonary vascular remodeling. Considering that NLRP3 had the strongest binding capacity with quercetin, many studies have emphasized that NLRP3 activation is closely associated with right ventricular function in PAH [42, 43]. Hence, we also examined the expression of NLRP3 inflammasome‐related factors. The results showed that safflower inhibited the activation of NLRP3 inflammasome in MCT‐induced PAH rats, and further in vitro experiments revealed that the efficacy of the NLRP3 inhibitor (MCC950) was comparable to that of safflower. Taken together, we speculate that safflower delays the progression of PAH mainly by inhibiting NLRP3 inflammasome.

In this study, we employed network pharmacology, molecular docking, and experimental validation for the first time to identify the targets and mechanism of safflower against PAH. These results may have implications for the use of TCM in the treatment of PAH. However, we must acknowledge the limitations of our research. The regulation of therapeutic targets by active compounds and the specific mechanisms of these targets in PAH has not been explored. Besides, the pharmacological activity of safflower involves multiple targets and pathways; thus, its application potential in clinical practice needs to be further confirmed.

## 5 Conclusion

In summary, our study offers preliminary evidence that safflower may ameliorate PAH through the suppression of NLRP3 inflammasome activation.

## Author Contributions

The authors confirm contribution to the paper as follows: conceptualization: A.C.; data curation, formal analysis, investigation, and methodology: J.C., L.Y., C.R., and F.H.; funding acquisition: S.D., J.C., and F.H.; project administration, resources, and supervision: J.C. and L.Y.; software: C.R. and F.H.; validation: S.D.; visualization and writing – original draft: S.D.; and writing – review and editing: A.C. All authors reviewed the results and approved the final version of the manuscript.

## Ethics Statement

The study followed the tenets of the Declaration of Helsinki and was approved by the Ethical Committee of Xiamen University (ethical approval code: XMULAC20230175‐1).

## Conflicts of Interest

The authors declare no conflicts of interest.

## Supporting information


**Figure S1** Identification of PASMCs and PAECs through immunofluorescence staining.

**Figure S2** HPLC chromatograms of hydroxysafflor yellow A and quercetin in SI.

## Data Availability

The data that support the findings of this study are available on request from the corresponding author. The data are not publicly available due to privacy or ethical restrictions.
