# Supplementary figures and images for: Safflower Alleviates Pulmonary Arterial Hypertension by Inactivating NLRP3: A Combined Approach of Network Pharmacology and Experimental Verification
Source: Clin Respir J. 2024 Aug 18;18(8):e13826. doi: 10.1111/crj.13826 (PMC11330698; doi:10.1111/crj.13826)

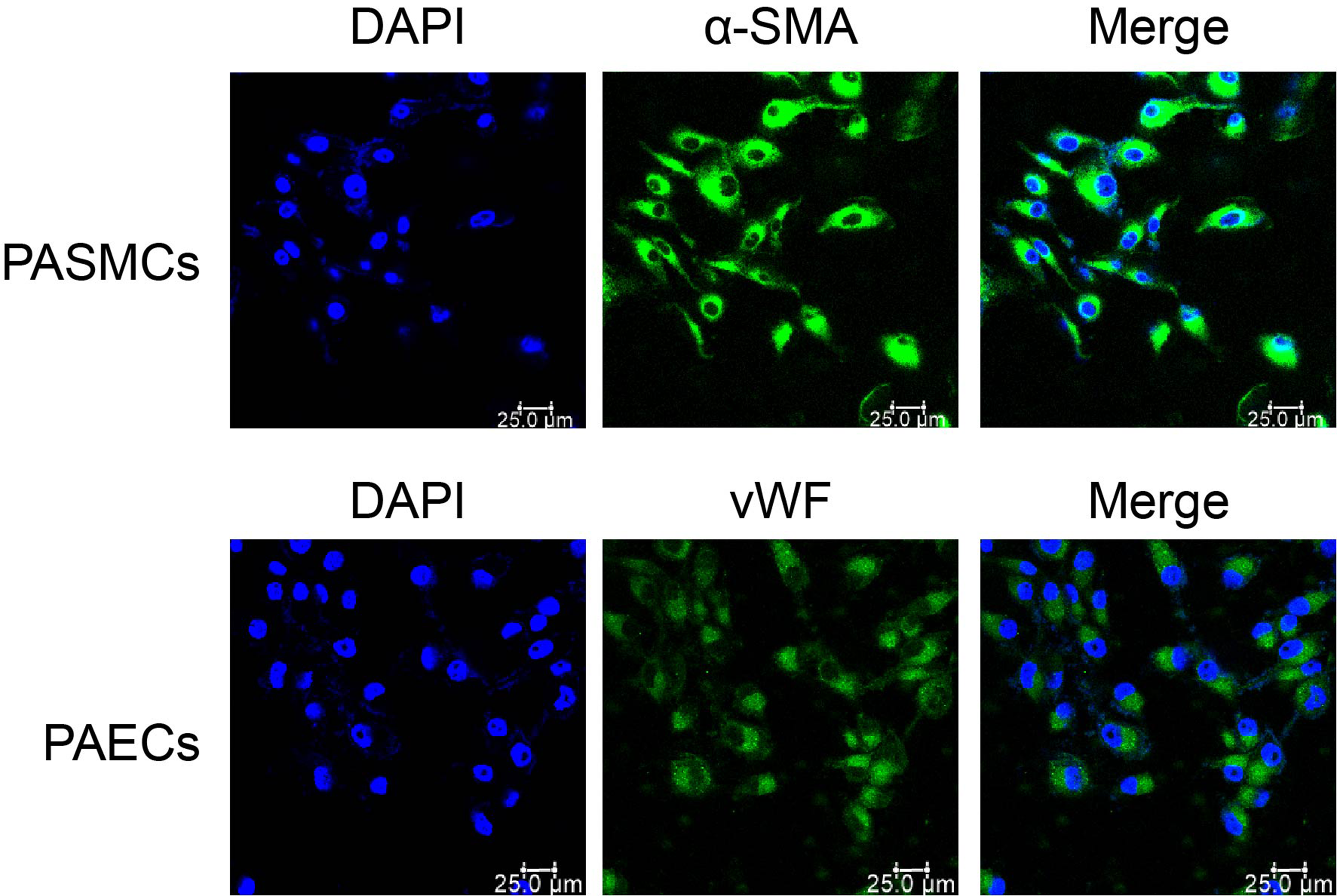

Supplement: Supplementary file 1 — Figure S1 Identification of PASMCs and PAECs through immunofluorescence staining. [file CRJ-18-e13826-s002.tif]

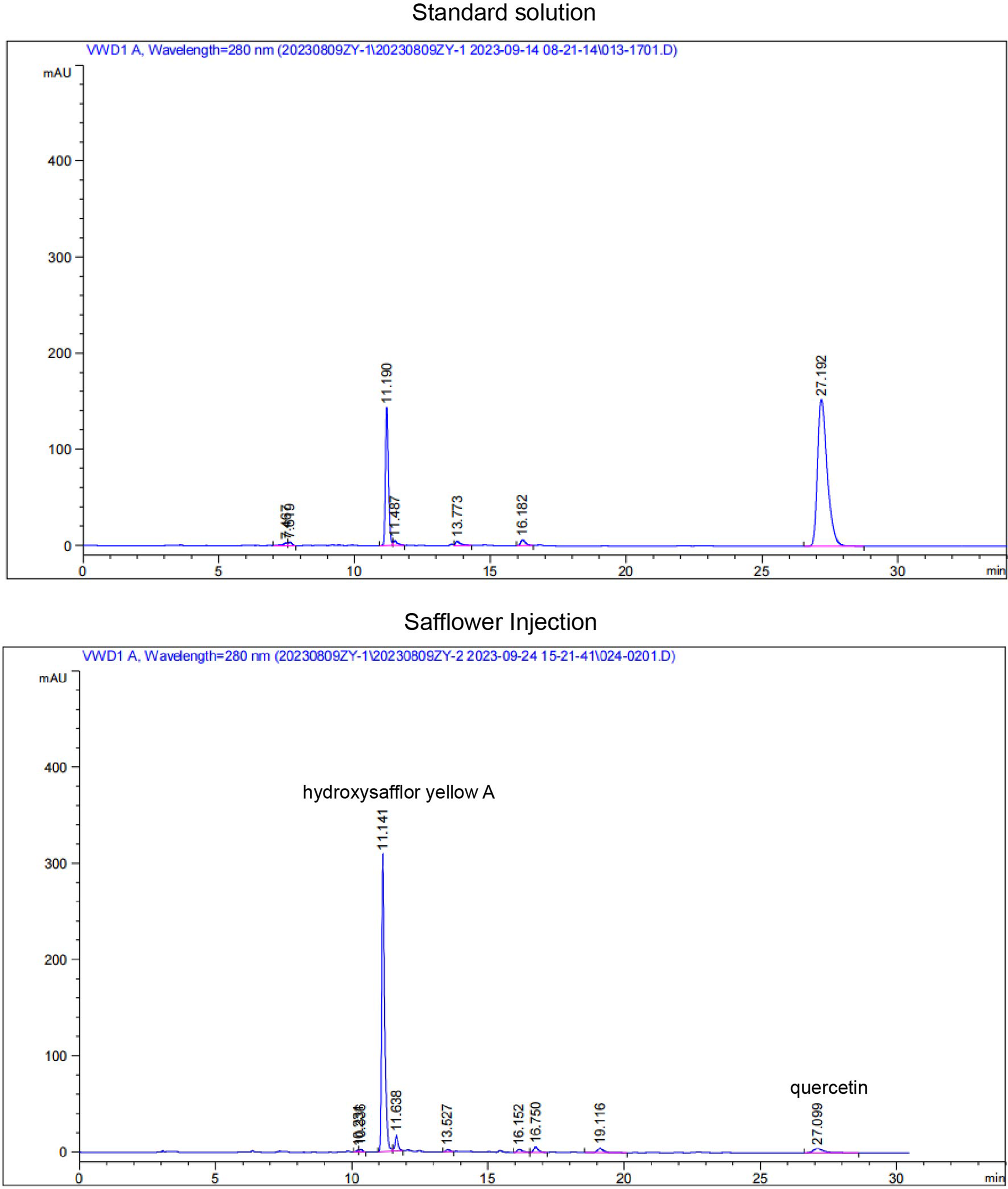

Supplement: Supplementary file 2 — Figure S2 HPLC chromatograms of hydroxysafflor yellow A and quercetin in SI. [file CRJ-18-e13826-s001.tif]
